# Oncological Outcomes of Open Versus Minimally Invasive Surgery for Ductal Adenocarcinomas of Pancreatic Head: A Propensity Score Matching Analysis

**DOI:** 10.3390/curroncol31100455

**Published:** 2024-10-11

**Authors:** Alessandro Giani, Michele Mazzola, Michele Paterno, Andrea Zironda, Pietro Calcagno, Emma Zuppi, Paolo De Martini, Giovanni Ferrari

**Affiliations:** Division of Minimally-Invasive Surgical Oncology, ASST Grande Ospedale Metropolitano Niguarda, 20162 Milano, Italy; a.giani3@gmail.com (A.G.); michele.paterno.94@gmail.com (M.P.); andrea.zironda@ospedaleniguarda.it (A.Z.); pietro.calcagno@ospedlaeniguarda.it (P.C.); emma.zuppi@unimi.it (E.Z.); paolo.demartini@ospedaleniguarda.it (P.D.M.); giovanni.ferrari@ospedaleniguarda.it (G.F.)

**Keywords:** minimally invasive pancreaticoduodenectomy, pancreatic resection, pancreatic ductal adenocarcinoma, radicality, R0, survival

## Abstract

Background: Minimally invasive pancreatic resections (MIPRs) have been shown to be safe and feasible, but there is still a lack of high-level evidence on oncological outcomes for cephalic pancreatic ductal adenocarcinoma (PDAC). The aim of this study was to compare the oncological outcomes of patients undergoing MIPR and open pancreatic resection (OPR) for pancreatic head cancer in a single high-volume center. Methods: Data from a prospectively collected database of patients who underwent radical-intent surgery for resectable and borderline resectable PDAC of the head at our institution between January 2013 and May 2023 were retrieved and analyzed, comparing the surgical and oncological outcomes of MIPR and OPR, using a propensity score matching analysis. Results: In the study period, 220 patients were selected. After matching, a total of 81 MIPRs and 81 OPRs were compared. No difference was found regarding R0 rate (OPR 83.9% vs. MIPR 74.1%, *p* = 0.122). Median overall survival (24 and 31 months for the OPR and MIPR groups, respectively; log rank *p* = 0.665) and disease-free survival (12 and 21 months for the OPR and MIPR groups, respectively; log rank *p* = 0.118) did not differ between the groups. The MIPR group was associated with a greater number of harvested lymph nodes (22 vs. 16, *p* = 0.0008), longer operative time (565 vs. 420 min, *p* < 0.0001), and shorter length of stay (12 vs. 18 days; *p* = 0.0001). No differences between the groups were found regarding all other postoperative and pathological outcomes. Conclusions: Regarding oncological outcomes, MIPR appeared to be comparable to OPR for treating patients with PDAC of the head. Despite an increased operative time, MIPR was associated with a greater number of LNs harvested and a shorter length of stay.

## 1. Introduction

Pancreatic ductal adenocarcinoma (PDAC) still represents a therapeutic challenge due to its poor prognosis, which will lead it to become the second leading cause of cancer-related death within the next decade [1]. Although multimodal strategies based on several advanced treatments, including perioperative chemotherapy, have been improved, surgery remains the only therapy with a potentially curative intent, and is the cornerstone to effectively improving the long-term survival for patients with PDAC [1,2].

Because of its earlier clinical onset, PDAC located in the head of the pancreas has a higher resectability rate than body and tail PDAC, but shows a more malignant phenotype, poorer differentiation, and a worse survival for all pathologic stages [3]. 

Patients with PDAC of the head undergoing resection, i.e., pancreaticoduodenectomy (PD) and total pancreatectomy (TP), have a higher risk of postoperative complications and mortality as compared with those undergoing left pancreatic resections, which can heavily affect overall prognosis [4].

Several retrospective studies have demonstrated that minimally invasive PD (MIPD) is not inferior to open PD (OPD) regarding short- and long-term outcomes [5,6]. However, the heterogeneity of indication for surgery, not limited to PDAC but also considering other periampullary tumors, and the inclusion of highly different-volume centers could limit the reproducibility and reliability of results [7].

The aim of this study was to compare the oncological outcomes of patients undergoing minimally invasive and open pancreatic resections (MIPRs and OPRs) for pancreatic head cancer in a single tertiary referral center.

## 2. Material and Methods

### 2.1. Study Overview

This is a retrospective cohort study based on a prospectively maintained database. All consecutive adult patients (age ≥ 18 years) undergoing radical-intent surgery for PDAC of the pancreatic head, defined as all PDACs involving pancreatic parenchyma on the right of the gastroduodenal artery, at our division (a tertiary referral center with an overall caseload of 60–80 pancreatic resections per year) between January 2013 and May 2023 were considered and analyzed with an intention-to-treat design. The exclusion criteria were locally advanced tumor [8], ASA 4, and concomitant non-pancreatic malignancies.

Since 2017, laparoscopy has been the preferred approach in all patients with indications for PD and TP, except for those with preoperatively known major vascular involvement, neoadjuvant treatment, and anesthesiologic contraindications to pneumoperitoneum. The robotic approach was introduced in our practice in September 2022, after an adequate learning curve in other fields of surgery (colorectal, oesophagogastric, and left pancreatic resections), and has rapidly become the only minimally invasive approach for pancreatic resections. The selection criteria for MIPR have gradually evolved over time to also include patients undergoing neoadjuvant treatment. 

The study protocol followed the ethical guidelines of the 1975 Declaration of Helsinki (as revised in Brazil 2013). The Local Ethical Committee review of the protocol deemed that formal approval was not required owing to the retrospective, observational, and anonymous nature of this study. The results are reported according to Strengthening the Reporting of Observational Studies in Epidemiology (STROBE) [9].

### 2.2. Surgical Technique and Perioperative Management

Patients were staged with serum carbohydrate antigen 19-9 (CA 19.9) and a computed tomography (CT) scan of the chest and the abdomen, and then evaluated by a multidisciplinary tumor board. Resectability and indication for neoadjuvant therapy were evaluated according to international guidelines [8,10]. At the end of the treatment, patients who had undergone neoadjuvant therapy were re-staged in order to rule out progressive disease and confirm surgical indication.

Perioperative care was similar in all patients, and followed ERAS recommendations [11]. 

The techniques adopted for OPD and laparoscopic PD were previously described [12,13]. For robotic procedures, a pure robotic approach (4 robotic trocars and 1 laparoscopic trocar) with a Da Vinci Xi system™ was used [14]. Both the approaches (open and minimally invasive) were performed following the same surgical steps. A complete dissection of the mesopancreas was performed according to the artery-first approach principle after an extensive Kocher maneuver. The gastric antrum or duodenum and the gastroduodenal artery were sectioned using staplers. The pancreatic neck was underpassed and divided using cold scissors, as well as the common hepatic duct. Frozen sectioning of the pancreatic and bile duct stump was always performed for confirming negative resection margins. The fistula risk score (FRS) was calculated [15]. Pancreaticojejunostomy was performed according to Cattell–Warren or Blumgart, depending on the surgeon’s preference and independently from the surgical approach [16,17]. A single-layer end-to-side hepaticojejunostomy was performed using running or interrupted sutures, depending on the diameter and thickness of the biliary duct. Gastro- and duodenojejunostomy were performed in an antecolic fashion, using a 60 mm linear stapler or manual double-layer handsewn, respectively. In all patients, one or two drains close to the pancreaticojejunostomy were positioned.

The nasogastric tube was removed immediately after surgery, with a near-zero fluid balance applied to avoid fluid overload. The patient was mobilized the day after surgery when feasible. The patient was fed with a liquid diet on the first operative day and a solid diet the day after, if tolerated. Drain management was based on a selective early removal policy, according to patients’ conditions and drain fluid characteristics [18]. 

### 2.3. Follow-Up 

Adjuvant treatment was indicated according to the international guidelines [19]. The outpatient oncological follow up was scheduled as follows: clinical evaluation, blood tests, and contrast-enhanced CT scan of the chest and the abdomen at one month after surgery and every six months within 5 years of follow-up. History, physical examination, and serum CA 19.9 levels were also checked at each follow-up visit. During adjuvant therapy, a contrast-enhanced CT scan of the chest and the abdomen was performed to evaluate the efficacy of chemotherapy every four cycles. Patient surveillance was closed in May 2024.

### 2.4. Variables and Definitions

Age, gender, body mass index (BMI), ASA score, age-adjusted Charlson comorbidity index (ACCI) [20], previous abdominal surgery, neoadjuvant therapy, preoperative disease assessment [8], suspected vascular involvement, preoperative biliary drainage, and serum CA 19.9 levels were recorded at presentation.

Surgery-related variables were the extent of resection (PD or TP), surgical approach (open, laparoscopic, or robotic), associated procedures, vascular resection, duration of surgery, blood loss, operative time, pancreatic texture, and Wirsung diameter. Conversion from a minimally invasive to open approach was considered when the resection or reconstruction phase needed to be completed by any type of laparotomy. Length of hospital stay was defined as the number of nights spent in the hospital from the day of the surgical procedure until discharge. Postoperative complications were recorded at 90 days and graded according to the Clavien–Dindo classification [21]. Complications graded as ≥ 3a were considered to be severe. Pancreas-specific complications were recorded separately in accordance with the recommendations by the International Study Group of Pancreatic Surgery, as follows: postoperative pancreatic fistula (POPF), postpancreatectomy hemorrhage (PPH), delayed gastric emptying (DGE), and bile leak (BL) [22,23,24]. Readmission rate and postoperative mortality were calculated at 90 days from surgery. 

Grading, pTNM, and pathologic stage were classified according to the American Joint Committee on Cancer staging system 8th edition [25]. The median number of lymph nodes (LNs) harvested and median number of positive LNs were collected. Resection margins were defined according to the Royal College of Pathologists; margin status was considered as R1 when the distance between the tumor and any resection margin was ≤1 mm [26].

The oncologic follow-up was updated to the latest outpatient visit or telephone interview. The type of adjuvant treatment and the chemotherapeutic regimen were recorded. The following data were also systematically collected: cancer recurrence, site of recurrence, and cancer-related death. Disease recurrence (distant or local) was diagnosed on the basis of clinical, radiological, and laboratory exams after local multidisciplinary tumor board discussion; histological confirmation was indicated when a clear diagnosis could not be obtained from the aforementioned techniques. Disease-free survival (DFS) was defined as the time interval expressed in months from surgery to any cancer recurrence. Overall survival (OS) was defined as the time interval in months from surgery to death; if the patient was alive, data were censored at the last available visit. 

### 2.5. Study Endpoints

The primary endpoint was R0 in the open (OPR) and minimally invasive (MIPR) pancreatic resection groups. The secondary endpoints were LNs harvested, OS, DFS, overall and pancreas-specific complication rates, length of hospital stay, and 90-day mortality.

### 2.6. Statistical Analysis

Continuous data are presented as median and IQRs, and categorical data as frequencies and proportions. The normal distribution of continuous variables was assessed with the Shapiro–Wilk test. Categorical variables were analyzed by the Fisher exact test or Chi-Square test and continuous variables by the Student’s *t*-test or Mann–Whitney test, as appropriate. To reduce the bias arising from selection and the lack of randomization, Propensity Score Matching (PSM) was run, with 1:1 nearest-neighbor matching and a caliper of 0.1 [27]. Significant variables (*p* < 0.05) at univariate analysis were used to run the matching, as follows: neoadjuvant therapy, extent of resection, tumor size, and TNM stage. OS and DFS were evaluated by the Kaplan–Meier method. Comparison among groups was performed with the log-rank test. The median follow-up time was estimated with the reverse Kaplan–Meier method. All the statistics were 2-tailed and statistical significance was accepted when *p* < 0.05. All the statistical analyses were performed using IBM SPSS Statistics version 26.0.0 (IBM corp.; Armonk, NY, USA).

## 3. Results

During the study period, a total of 232 patients underwent an elective curative-intent pancreatic resection for head cancer. Among them, 12 patients met the exclusion criteria due to a locally advanced extension of their tumor, thus, 220 patients were finally included in the analysis, with 139 in the OPR group and 81 in the MIPR group.

The groups did not differ in terms of baseline characteristics, except for the rate of patients undergoing neoadjuvant therapy (19.4% vs. 8.6%, *p* = 0.032, respectively), those with suspected preoperative vascular involvement (31.7% vs. 14.8%, *p* = 0.005, in OPR and MIPR groups, respectively), and tumor size (30 [IQR 23–35] mm vs. 25 [IQR 20.5–30] mm, *p* = 0.032), which were in favor of the OPR group. In the whole cohort, 28 patients underwent unplanned total pancreatectomy, 23 (16.5%) in the OPR group and 5 (6.2%) in the MIPR group (*p* = 0.026), due to infiltration of the pancreatic margin at the frozen section (12 patients) and very-high-risk pancreaticojejunostomy associated with concomitant relevant patient comorbidities (16 patients).

In order to reduce the impact of treatment selection bias on the estimation of causal treatment effects, PSM was performed based on the variables which differed between the groups at baseline and those deemed to affect long-term outcomes (neoadjuvant therapy, preoperative vascular involvement, type of surgery, tumor size, and TNM stage). After PSM, no difference was found between the groups, which consisted of 81 patients each, except for the loss of previous comparability in terms of tumor grading. Baseline characteristics before and after PSM are summarized in Table 1.

### 3.1. Surgical Outcomes

Surgical outcomes after PSM are reported in Table 2. In the MIPR group, 48 patients (59.3%) received a laparoscopic approach and 33 patients (40.7%) a robotic approach; a subanalysis between these minimally invasive approaches was performed and the results are listed in Supplementary Tables S1–S3. The conversion rate was 6.2% (5/81), mostly due to vascular invasion (40%) rather than adhesion (20%), and intraoperative bleeding (20%). Pylorus-preserving resection was performed in 0 (0.0%) and 27 (25.9%) patients (*p* < 0.001) in the OPR and MIPR groups, respectively. The OPR group showed a significantly lower median operative time (420 [IQR 357.5–470] min vs. 565 [IQR 510–607.5] min, *p* < 0.0001), but a longer hospital stay (18 [IQR 15–27] days vs. 12 [IQR 8–18] days, *p* = 0.0001) and a worse trend for overall complications (58% vs. 43.2%, *p* = 0.059) in comparison with the MIPR group. No differences between the groups were found regarding all other postoperative outcomes.

### 3.2. Oncological Outcomes

Oncological outcomes after PSM are reported in Table 3. The primary endpoint (R0 rate) did not differ between the OPR and MIPR groups (83.9% vs. 76.5%, *p* = 0.236), and nor did the site of positive margin (*p* = 0.552). The MIPR group showed a greater number of LNs harvested (22 [IQR 15–27] vs. 16 [IQR 12–22], *p* = 0.0008), with no difference between the groups regarding the median number of positive LNs (*p* = 0.877) and LNs ratio (*p* = 0.149).

The rate of patients receiving adjuvant therapy was similar between the groups (OPR 63% vs. MIPR 56.8%, *p* = 0.423), as was the type of treatment received (chemotherapy or chemo-radiotherapy, *p* = 0.361). The chemotherapy protocols administered differed between the groups, with a higher administration of Gemcitabine in the OPR and of FOLFIRINOX in the MIPR group (*p* < 0.0001). 

The median follow-up was 52 months in the entire population (IQR 18–116), with 98 months (IQR 62–140) and 24 months (IQR 13–48) for the OPR and MIPR groups, respectively (*p* < 0.001). During the follow-up period, 82 patients (50.6%) were diagnosed with a recurrence and 96 (59.3%) were deceased. Distant recurrence was observed in 54 patients (33.3%); among these, 5 patients (3.1%) had multiple sites of distant recurrence at the time of diagnosis. Liver was the most frequent site of metastases (17.9%), followed by lung (16.1%), and distant LNs (1.2%). Local recurrence was diagnosed in 28 patients (17.3%).

**Figure 1:**
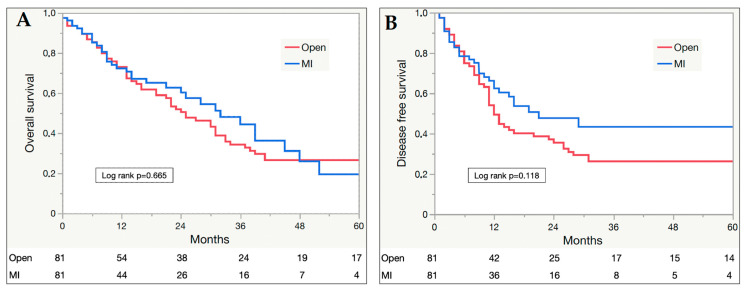
Kaplan–Meier plots of 5-year overall survival (**A**) and disease-free survival (**B**) for open and minimally invasive (MI) resections.

The median OS for the entire cohort was 27 months (24 and 31 months for the OPR and MIPR groups, respectively). No difference in 5-year OS (log rank *p* = 0.665) and DFS (log rank *p* = 0.118) was found between the groups. In particular, 1-, 3-, and 5-year OS were 73.0%, 34.3%, and 26.5% in the OPR and 72.3%, 48.1%, and 19.4% in the MIPR group, respectively. Furthermore, 1-, 3-, and 5-year DFS were 54.1%, 26.3%, and 26.3% for the OPR group and 66.2%, 43.4%, and 43.4% for the MIPR group. Kaplan–Meier curves are depicted in Figure 1.

## 4. Discussion

The present PSM cohort study demonstrated no difference in terms of R0 resection rate, survival outcomes, and postoperative complications between MIPR and OPR for treating patients with PDAC of the head. Furthermore, MIPR was associated with a longer operative time, but a shorter length of hospital stay and a greater number of LNs harvested.

The application of minimally invasive approaches to pancreatic resection is no longer pioneering. Thanks to their gradual and safe implementation over the last decades, MIPRs have become the standard of care for treating benign, low-grade, and even malignant tumors [14,28]. Safety and feasibility have generally been proven, not only for left pancreatic resections, but even for more challenging procedures such as PD and TP [29,30]. However, doubts still exist about the oncological safety of MIPR due to the lack of high-level evidence, especially regarding PD. The non-inferiority of MIPR for treating patients with pancreatic cancer located in the body and tail was proven by a recent randomized clinical trial [31]. Several high-quality studies have been published that report promising results in terms of surgical and oncological outcomes by using MIPD in comparison with OPD, but including heterogeneous patient cohorts in terms of indication for surgery [32]. Periampullary neoplasm is a common definition for different tumors originating around the same anatomical region, the ampulla of Vater. Although resection of the duodenum and pancreatic head represents the curative surgical treatment for all these tumors, independently from their histotype, relevant differences have been shown in terms of prognosis, with a 5-year survival ranging from 15 to 59% [33,34]. PDAC is the most frequent type of periampullary cancer and shows specific features such as a lower resectability rate, more advanced stage at diagnosis, and particular tendency to infiltrate the nerve sheaths, resulting in a worse prognosis and more challenging surgery [35,36]. 

The achievement of negative margins (R0) is the ideal goal of radical surgery, and it is particularly important when treating PDAC, since it represents a crucial factor associated with patient prognosis [37]. Large retrospective studies based on national registries reported no difference between MIPD and OPD in terms of R0 rate, with margin positivity ranging from 15 to 21% and from 16 to 26%, respectively; however, they also included 11–15% of patients with tumors other than PDAC, operated in highly different-volume centers, without a shared protocol for specimen evaluation [7,38,39]. In a previous study, we found a trend of a lower R0 rate for PDAC after MIPR as compared to other periampullary tumors, reflecting the location, usually closer to the resection margins, and biological behavior, with a tendency of PDAC to invade neural, vascular, and surrounding vital structures [40,41]. A recent meta-analysis showed a higher rate of R0 resection after MIPD in comparison to OPD [42]. Since this result could be influenced by selection bias, preferring minimally invasive approaches for patients with smaller tumors and at earlier stages, a PSM analysis was adopted in the present study in order to balance the baseline characteristics of patients, including variables known to affect resection margin status such as neoadjuvant therapy, vascular involvement, type of surgery, tumor size, and stage. Our results showed no difference between the groups regarding R0 rate, even though a trend in favor of OPR could be seen, probably due to the higher proportion of less differentiated PDACs in the MIPR group after PSM. The rate of microscopic positive margin in the present study was 23.5% after MIPR, consistent with previous publications reporting rates which reached 30–45% for patients with PDAC, especially when using a strict pathology protocol, with R1 considered for tumors at a distance of ≤1 mm from resection margins [43,44]. Several studies investigating oncological outcomes after MIPR did not specify the site of positive margins; our study showed no difference in both the R1 rate and site of positive margin when comparing MIPR and OPR. Most patients were considered R1 at the posterior margin, which was defined as a mobilization margin, that is, where two adjacent organ surfaces have simply been separated by developing embryological planes; this outcome is not modifiable by a more radical or aggressive approach, but simply reflects the position of the tumor itself, and does not appear to affect survival [45]. The second most common positive margin was the medial one, namely, the vascular groove margin; the absence of differences in the R1 rate for this margin between MIPR and OPR may be explained by the adoption, in both approaches, of the same surgical technique based on an artery-first approach, which was associated with better results in terms of resection margins [46]. 

The LN status is an important predictor of survival for patients with PDAC; standard lymphadenectomy, aiming to retrieve at least 15 LNs, is recommended for adequate nodal staging [47]. In a network meta-analysis comparing OPD, laparoscopic PD, and robotic PD, no difference was seen in the number of harvested LNs among the different approaches; the authors reported a potential selection bias in using MIPD for treating non-malignant lesions and adopting different pathologic processing techniques and definitions, which can affect the LN count [48]. A similar result was reported in a meta-analysis focusing specifically on PDAC treatment, where no difference was found between MIPD and OPD regarding the number of LNs harvested [42]. In the present study, MIPR was associated with a greater number of LNs retrieved, even when obtaining an adequate number of LNs by using OPR (22 vs. 16, *p* < 0.001); however, no difference between the groups emerged regarding LNs ratio. Since no difference was found in the number of harvested LNs when comparing laparoscopic and robotic resections (data of the sub-analysis are reported in the Supplementary Materials), the better outcomes after MIPR in comparison to OPR may be explained through the advantages of minimally invasive technology, allowing for magnified visualization and a more precise dissection combined with the systematic use of an artery-first approach.

Although the importance of histopathological outcomes was largely proven as a prognostic factor for patients with PDAC, they represent a surrogate of the most important oncological outcomes, namely patient survival and disease recurrence. Due to the relatively recent introduction of MIPR in the treatment of PDAC, high-quality survival data from randomized clinical trials are still lacking, with those from retrospective trials reporting a median follow-up ranging between 11 and 22 months or not reporting it at all in the majority of studies [42,49,50]. The median follow-up for the whole cohort of the present study was 52 months, ranging from 98 to 24 months in the OPR and MIPR groups, respectively (*p* < 0.001), due to the more recent introduction of MIPR into clinical practice. Considering that the median OS and time to first recurrence reported in the literature for patients with resected PDAC, regardless of the surgical approach, varied between 20 and 28 months and 8 and 13 months, respectively, the median follow-up of the present study may ensure reliable data for both patients undergoing OPR and MIPR [51,52]. Some studies omitted data from patients operated on during the initial phase of the learning curve for MIPR, introducing a relevant selection bias; on the contrary, in the present study, all patients undergoing MIPR were included, since the effect of the learning curve could influence other outcomes, such as the duration of surgery or blood loss, but never compromise oncological ones [41,42]. As previously reported, acceptable results can be achieved by experienced surgeons, skilled in both advanced minimally invasive and open pancreatic surgery, even during their early learning curve for MIPR [16]. A recent meta-analysis found no difference in the OS between patients undergoing laparoscopic PD and OPD for treating PDAC, and a comparable DFS and OS were observed also for patients undergoing robotic PD and OPD [53,54]. The non-inferiority of MIPD as compared to OPD in terms of survival outcomes was also confirmed in the setting of patients undergoing neoadjuvant treatments [38]. Consistently, in the present study, survival outcomes did not differ between MIPR and OPR, neither in terms of 5-year OS (log rank *p* = 0.665) nor DFS (log rank *p* = 0.118), with a median OS for the entire cohort of 27 months.

Adjuvant therapy is a strong prognostic factor for improving both OS and DFS in patients with PDAC [51,55]. The rate of patients receiving adjuvant therapy varies widely in the literature; national registry data reported an adherence to adjuvant chemotherapy of around 56–60%, regardless of the surgical approach received, but in other studies, adjuvant chemotherapy was administered in nearly 70% of patients [7,56,57]. Several factors were associated with receiving adjuvant therapy, such as patients’ age and comorbidity and postoperative complications [57]. In the current study, the overall and pancreas-specific complication rates in the whole cohort were consistent with the literature, without differences between the groups, except for a shorter length of hospital stay in favor of MIPR [32,50]. However, patients’ age and CCI were higher compared with the literature, explaining the slightly lower rate of the administration of adjuvant therapy (nearly 50%). The groups differed concerning the regimen of adjuvant chemotherapy received, with the prevalence of a Gemcitabine-based protocol in the OPR group and one of FOLFIRINOX in the MIPR group. These results should be considered in light of the evolving evidence, showing a benefit for patients treated with FOLFIRINOX rather than Gemcitabine, albeit a more severe toxicity [58]. Further studies are needed to explore the best chemotherapeutic drug combination for each patient and whether minimally invasive surgery could contribute to increasing the rate of patients able to tolerate more severe chemotherapy regimens by improving their postoperative functional recovery. However, in the current study, the groups did not show differences in terms of postoperative outcomes, except for a trend toward a lower overall complication rate in favor of the MIPR group; this could have been due to the difficulty in identifying the short-term benefits of minimally invasive approaches using a retrospective study design.

The present study presents several limitations. Although minimized by the PSM analysis, the results of this single-center retrospective series may be affected by several unidentified confounders. A greater number of patients in the MIPR group received pylorus-preserving resection in comparison to the OPR group; this could have affected postoperative outcomes, especially DGE. However, according to the literature, no difference was found between the groups in terms of postoperative complications, confirming the absence of a clear superiority of one reconstructive technique over the other [58,59]. The results could have been influenced by changes over time in perioperative management, surgical experience, and different chemotherapy regimens applied. Although laparoscopic and robotic pancreatic resections showed similar surgical outcomes, robotic pancreatic resection has become the standard approach since September 2022 due to the advantages perceived by surgeons in terms of better vision, ease of instrument handling, and ergonomics; however, a more accurate analysis of the differences between the laparoscopic and robotic approaches is beyond the scope of this study. Nonetheless, while patients operated on in the early adoption phase of MIPR were included, the learning curve for OPR had already been achieved at the beginning of this study, potentially affecting the results in favor of OPR. Finally, the relatively small sample size could potentially generate a type II error. 

## 5. Conclusions

Based on our PSM cohort study, regarding oncological outcomes, MIPR appeared to be comparable to OPR for treating patients with PDAC of the head. Despite an increased operative time, MIPR was associated with a greater number of LNs harvested and a shorter length of stay. Considering the retrospective nature of this study, firm conclusions cannot be drawn and future high-quality trials are needed.

## Figures and Tables

**Table 1:** Baseline characteristics of patients undergoing OPR and MIPR, before and after PSM.

Characteristic	Original Cohort		*p*	Matched Cohort		*p*
	OPR (139)	MIPR (81)		OPR (81)	MIPR (81)	
Sex (Female)	74 (53.2)	44 (54.3)	0.876	45 (55.6)	44 (54.3)	0.874
Age (years)	72 (61–77)	71 (62.5–78)	0.347	73 (62.5–77)	71 (62.5–78)	0.504
BMI (kg/m^2^)	23.9 (22.0–26.0)	24.7 (22.5–27.4)	0.118	24 (22.0–26)	24.7 (22.5–27.4)	0.136
ACCI	5 (4–6)	5 (4–6)	0.545	5 (4–6)	5 (4–6)	0.527
ASA			0.116			0.586
ASA 1	13 (9.3)	3 (3.7)		6 (7.4)	3 (3.7)	
ASA 2	93 (66.9)	64 (79.0)		62 (76.5)	64 (79.0)	
ASA 3	33 (23.7)	14 (17.3)		13 (16.1)	14 (17.3)	
Previous abdominal surgery	51 (36.7)	30 (37.0)	0.959	22 (27.2)	30 (37.0)	0.178
Neoadjuvant therapy	27 (19.4)	7 (8.6)	0.032	8 (9.9)	7 (8.6)	0.786
Preoperative radiological vascular involvement	44 (31.7)	12 (14.8)	0.005	20 (24.7)	12 (14.8)	0.114
Hepatic vascular anomalies	36 (25.9)	21 (25.9)	0.996	19 (23.5)	21 (25.9)	0.715
Preop biliary drainage	75 (54.0)	37 (45.7)	0.236	47 (58.0)	37 (45.7)	0.115
Preoperative CA 19.9	171.2 (34.9–508)	124.5 (15.8–385)	0.148	204.7 (27.5–536.6)	124.5 (15.8–385)	0.109
Type of surgery			0.026			0.754
PD	116 (83.4)	76 (93.8)		75 (92.6)	76 (93.8)	
TP	23 (16.5)	5 (6.2)		6 (7.4)	5 (6.2)	
Vascular resection			0.765			0.589
PV	2 (1.4)	2 (2.5)		1 (1.2)	2 (2.5)	
SMV	5 (3.6)	4 (4.9)		2 (2.5)	4 (4.9)	
Grading			0.075			0.0001
G1 (well)	11 (7.9)	2 (2.5)		10 (12.3)	2 (2.5)	
G2 (moderately)	82 (59.0)	42 (51.8)		57 (70.4)	42 (51.8)	
G3 (poorly)	46 (33.1)	37 (45.7)		14 (17.3)	37 (45.7)	
Tumor size (mm)	30 (23–35)	25 (20.5–30)	0.032	27 (22–31)	25 (20.5–30)	0.422
pT			0.193			0.494
pT1	22 (15.8)	19 (23.5)		13 (16.0)	19 (23.5)	
pT2	96 (69.1)	55 (67.9)		60 (74.1)	55 (67.9)	
pT3	21 (15.1)	7 (8.6)		8 (9.9)	7 (8.6)	
pN+	95 (68.4)	48 (59.3)	0.173	51 (63.0)	48 (59.3)	0.628
pN			0.364			0.727
pN0	44 (31.6)	33 (40.7)		30 (37.0)	33 (40.7)	
pN1	60 (43.2)	32 (39.5)		37 (45.7)	32 (39.5)	
pN2	35 (25.2)	16 (19.8)		14 (17.3)	16 (19.8)	
Wirsung diameter (mm) *	3 (2–5)	4 (2–5)	0.557	3 (2–5)	4 (2–5)	0.071
FRS *	2 (1–5)	3 (1–5)	0.479	3 (1–5)	3 (1–5)	0.428
Class of FRS *			0.261			0.163
Negligible (0 pts)	25 (21.6)	14 (18.4)		11 (14.7)	14 (18.4)	
Low (1–2 pts)	37 (31.9)	23 (30.3)		22 (29.3)	23 (30.3)	
Moderate (3–6 pts)	50 (43.1)	31 (40.8)		40 (53.3)	31 (40.8)	
High (7–10 pts)	4 (3.4)	8 (10.5)		2 (2.7)	8 (10.5)	

Variables are reported as number and percentage or median and interquartile range, as appropriate. OPR: open pancreatic resection, MIPR: minimally invasive pancreatic resection, PSM: Propensity Score Matching, BMI: body mass index, ACCI: Age-adjusted Charlson Comorbidity Index, ASA: American Society of Anesthesiologists Physical Status Classification System, CA 19.9: Carbohydrate Antigen 19.9, PD: pancreaticoduodenectomy, TP: total pancreatectomy PV: portal vein, SMV: superior mesenteric vein, and FRS: Fistula Risk Score. * Variable was calculated among patients undergoing pancreaticoduodenectomy. For pTNM stage the 8th edition of the AJCC TNM staging system was used.

**Table 2:** Surgical outcomes of patients undergoing OPR and MIPR, after PSM.

Characteristic	OPR (81)	MIPR (81)	*p*
Operative time	420 (357.5–470)	565 (510–607.5)	<0.0001
Surgical approach			
Laparoscopy	-	48 (59.3)	
Robot	-	33 (40.7)	
Conversion to open surgery		5 (6.2)	
Cause of conversion			
Adhesions		1/5 (20.0)	
Bleeding		1/5 (20.0)	
Vascular invasion		2/5 (40.0)	
Other		1/5 (20.0)	
Degree of stomach resection			0.0001
Pylorus-preserving	0	27 (33.3)	
Classic Whipple	81 (100.0)	54 (66.7)	
Blood loss (ml)	290 (200–340)	300 (200–575)	0.149
Length of stay (day)	18 (15–27)	12 (8–18)	0.0001
Overall complications	47 (58.0)	35 (43.2)	0.059
Reoperation	3 (3.7)	6 (7.4)	0.303
Necessity of ICU	9 (11.1)	8 (9.9)	0.798
Severe complications	18 (22.2)	19 (23.5)	0.851
Complications C-D:			0.587
1	44 (54.3)	47 (58.0)	
2	19 (23.5)	15 (18.5)	
3a	9 (11.1)	8 (10.0)	
3b	0 (0)	3 (3.7)	
4	4 (4.9)	4 (4.9)	
90-day mortality	5 (6.2)	3 (3.7)	0.468
CCI	8.7 (0–26.2)	0 (0–25.7)	0.218
Postoperative RBC transfusion	14 (17.3)	21 (26.3)	0.181
POPF *	11 (14.7)	7 (9.2)	0.301
POPF grade *			0.146
Grade B	9 (12.0)	3 (3.9)	
Grade C	2 (2.7)	4 (5.3)	
DGE	19 (23.5)	12 (14.8)	0.162
DGE grade			0.069
Grade A	12 (14.8)	5 (6.2)	
Grade B	4 (4.9)	7 (8.6)	
Grade C	3 (3.7)	0 (0)	
BL	4 (4.9)	6 (7.1)	0.513
BL			0.567
Grade A	0 (0)	2 (2.5)	
Grade B	3 (3.7)	3 (3.7)	
Grade C	1 (1.2)	1 (1.2)	
PPH	6 (7.4)	9 (11.1)	0.416
PPH grade			0.243
Grade A	1 (1.2)	1 (1.2)	
Grade B	5 (6.2)	4 (4.9)	
Grade C	0 (0)	4 (4.9)	
90-day readmission	2 (2.5)	9 (8.6)	0.086

Variables are reported as number and percentage or median and interquartile range, as appropriate. OPR: open pancreatic resection, MIPR: minimally invasive pancreatic resection, PSM: Propensity Score Matching, ICU: intensive care unit, C-D: Clavien–Dindo classification, CCI: Comprehensive Complication Index, RBC: red blood cells, POPF: Postoperative Pancreatic Fistula, DGE: Delayed Gastric Emptying, BL: Biliary Leak, and PPH: Post-Pancreatectomy Hemorrhage. * Variable was calculated among patients undergoing pancreaticoduodenectomy.

**Table 3:** Oncological outcomes of patients undergoing OPR and MIPR, after PSM.

Characteristic	OPR (81)	MIPR (81)	*p*
LNs harvested	16 (12–22)	22 (15–27)	<0.001
Positive LNs	1 (0–3)	1 (0–3)	0.877
LN ratio	0.07 (0–0.17)	0.04 (0–0.12)	0.149
R0	68 (83.9)	62 (76.5)	0.236
Site of positive margin			0.552
Posterior	8/13 (61.5)	15/19 (78.9)	
Medial	4/13 (30.8)	3/19 (15.8)	
Anterior	1/13 (7.7)	1/19 (5.3)	
Performed adjuvant therapy	51 (63.0)	46 (56.8)	0.423
Type of adjuvant therapy			0.361
Chemotherapy	37/51 (72.6)	37/46 (80.4)	
Chemo-radiotherapy	14/51 (27.4)	9/46 (19.6)	
Adjuvant therapy protocol			<0.001
mFOLFIRINOX	0 (0)	23/41 (56.1)	
Gem	40/50 (80.0)	5/41 (12.2)	
Gem + Capecitabine	5/50 (10.0)	10/41 (24.4)	
Other	5/50 (10.0)	3/41 (7.3)	

Variables are reported as number and percentage or median and interquartile range, as appropriate. OPR: open pancreatic resection, MIPR: minimally invasive pancreatic resection, PSM: Propensity Score Matching, mFOLFIRINOX: modified FOLFIRINOX, and Gem: Gemcitabine. Data about adjuvant therapy concerned patients still alive more than 90 days after surgery.

## Data Availability

The Data presented in the study are available on request from the corresponding authors.

## Supplementary Materials

The following supporting information can be downloaded at: https://www.mdpi.com/article/10.3390/curroncol31100455/s1, Table S1: Baseline characteristics of patients undergoing LPR and RPR; Table S2: Surgical outcomes of patients undergoing LPR and RPR; Table S3: Oncological outcomes of patients undergoing LPR and RPR; Table S4: Comparison of mean scores of the EORTC PATSAT-C33 scales of treatment satisfaction of caregivers at last assessment regarding the independent factors sex, age at diagnosis, Karnofsky Performance Scale and psychosocial distress of the patients (n = 61); Figure S1: Consort flow of depicting the inclusion criteria (n = 61); Figure S2: Distribution of applied questionnaires during the study period (n = 61).
